# Patient and physician perspectives of a smartphone application for depression: a qualitative study

**DOI:** 10.1186/s12888-021-03064-x

**Published:** 2021-01-29

**Authors:** Marie-Camille Patoz, Diego Hidalgo-Mazzei, Olivier Blanc, Norma Verdolini, Isabella Pacchiarotti, Andrea Murru, Laurent Zukerwar, Eduard Vieta, Pierre-Michel Llorca, Ludovic Samalin

**Affiliations:** 1 Department of Psychiatry, CHU Clermont-Ferrand, University of Clermont Auvergne, EA 7280 Clermont-Ferrand, France; 2 Bipolar and Depressive Disorders Unit, Hospital Clinic, University of Barcelona, Institute of Neuroscience, IDIBAPS, CIBERSAM, 170 Villarroel st, 12-0, 08036 Barcelona, Catalonia Spain; 3 Fondation FondaMental, Hôpital Albert Chenevier, Pôle de Psychiatrie, Créteil, France; 4 Clinique Mon Repos, Ecully, France

**Keywords:** Smartphone, Mobile health, Application, Depression, Major depressive episode

## Abstract

**Background:**

Despite an increasing number of smartphone apps, such therapeutic tools have not yet consistently demonstrated their efficacy and many suffer from low retention rates. To ensure the development of efficient apps associated with high adherence, we aimed to identify, through a user-centred design approach, patient and physician expectations of a hypothetical app dedicated to depression.

**Methods:**

We conducted semi-structured interviews with physicians (psychiatrists and general practitioners) and patients who had experienced a major depressive episode during the last 12 months using the focus group method. The interviews were audio recorded, transcribed and analysed using qualitative content analysis to define codes, categories and emergent themes.

**Results:**

A total of 26 physicians and 24 patients were included in the study. The focus groups showed balanced sex and age distributions. Most participants owned a smartphone (83.3% of patients, 96.1% of physicians) and were app users (79.2% of patients and 96.1% of physicians).

The qualitative content analysis revealed 3 main themes: content, operating characteristics and barriers to the use of the app. Expected content included the data collected by the app, aiming to provide information about the patient, data provided by the app, gathering psychoeducation elements, therapeutic tools and functionalities to help with the management of daily life and features expected for this tool. The “operating characteristics” theme gathered aims considered for the app, its potential target users, considered modalities of use and considerations around its accessibility and security of use. Finally, barriers to the use of the app included concerns about potential app users, its accessibility, safety, side-effects, utility and functioning. All themes and categories were the same for patients and physicians.

**Conclusions:**

Physician and patient expectations of a hypothetical smartphone app dedicated to depression are high and confirmed the important role it could play in depression care. The key points expected by the users for such a tool are an easy and intuitive use and a personalised content. They are also waiting for an app that gives information about depression, offers a self-monitoring functionality and helps them in case of emergency.

**Supplementary Information:**

The online version contains supplementary material available at 10.1186/s12888-021-03064-x.

## Background

Depression is a common psychiatric disorder with a prevalence reaching more than 300 million people around the world [1]. Its impact on patients’ psychosocial functioning and quality of life makes it a leading cause of disability worldwide [2, 3]. Despite the existence of effective treatments, such as antidepressants and cognitive-behavioural therapy, almost half of patients with depression stay untreated [4]. The appearance of computers and internet development in the ‘90s brought about the idea that technological devices could be used as therapeutic tools [5–8] so as to improve treatment rates [9].

In recent decades, the development of new technologies and their worldwide spread have led the way to new therapeutic and screening tools in mental health [10]. This field, called “mobile health” or “m-health” [11, 12], has seen exponential growth with more than 10,000 downloadable mental health smartphone applications (apps) associated with the extensive use of wearables such as smartbands or smartwatches [13]. Although m-health is a promising field for increasing access to mental health programmes, their current use in clinical practice is limited and most of the available apps have not yet consistently demonstrated their effectiveness in the management of depression [14]. Several causes may be considered regarding these two issues. First, most of the apps for depression fail to incorporate evidence-based practices or clinical expertise into their design [15–18]. Conversely, most of the scientifically validated apps are not available in apps stores [16, 19, 20]. Finally, m-health suffers from a low retention rate and engagement by users [21, 22] and is rarely integrated into clinical practice, relegating apps to a “self-medication tools” status [23, 24].

To overcome these issues, it could be of interest to implement a user-centred design approach to develop m-health tools to remain as close as possible to patient and physician expectations; this would facilitate both improved retention rates and app implementation in professional healthcare practice [21, 23].

Qualitative analysis is commonly used to assess patients’ expectations in various domains. It has mostly been used to evaluate patients’ use of pre-existing apps for depression [25, 26] or to explore the expectations of an app for depression in young people [27, 28]. To the best of our knowledge, no qualitative study has investigated both patient and physician expectations of a smartphone app dedicated to depression including patients with previous major depressive episodes (MDE), general practitioners and psychiatrists.

## Methods

### Study design

The perceptions and expectations of patients, general practitioners and psychiatrists concerning a hypothetical smartphone app dedicated to depression were investigated by using a qualitative design with a focus group methodology. The focus group method has been chosen because it is a reliable way to assess the participants’ expectations by facilitating the sharing of ideas and experiences among them.

The focus groups were conducted between November 2018 and May 2019 in France. Patients and physicians were allocated to separate groups. Psychiatrists and general practitioners were distributed randomly in the physicians groups.

### Sample and recruitment

Patients included were adults with a diagnosis of MDE in the last 12 months according to the Diagnostic and Statistical Manual of Mental Disorders - 4th edition, Text Revision (DSM-IV-TR) criteria [29]. They were required to understand and be fluent in French.

Physicians were psychiatrists or general practitioners working in the private and/or public sectors and dealing with patients with MDE in their clinical practice.

Eligible participants (patients and physicians) were screened by the investigator centres hosted by academic Departments of Psychiatry (Clermont-Ferrand, Lyon, Grenoble).

Physicians were solicited by email or by phone to participate.

Patients were recruited among in- and outpatient services of investigator centres. Those who gave consent to be contacted were followed up to arrange participation. The focus groups were held in the centre where the participants were recruited.

The study was carried out in accordance with ethical principles for medical research involving humans (WMA, Declaration of Helsinki). The assessment protocol was approved by the relevant ethical review board (CPP EST I, 2018-A01469–46). All subjects provided written informed consent to participate.

### Data collection

After a literature search on m-health and smartphone apps for depression, mirrored semi-structured interview guides have been established for patients and physicians (Additional file 1).

Before starting the session, patients completed a survey with their sociodemographic information, including age, gender and living place (urban or rural). They also indicated their smartphone and app use habits. Finally, the severity of their depressive symptoms was measured using the Inventory of Depressive Symptomatology (IDS-SR) questionnaire. The IDS-SR is a 30-item self-rated questionnaire assessing all the criterion symptom domains designated by the DSM-IV-TR [30].

Physicians’ age, gender, type of practice (public or private), number of visits per week, number of patients with depression seen each week and smartphone and app use habits were assessed through a questionnaire.

Each focus group included 6 to 8 participants and lasted from 60 to 90 min. There was an interviewer and an observer present for each group (LS, MCP or OB*),* all familiar with and well trained in the focus group methodology*.* All focus groups were audio recorded and transcribed verbatim.

### Analysis

Data collection and analysis were conducted at the same time in accordance with established qualitative methodologies [31]. After a focus group was transcribed verbatim, it was fully read then independently and manually coded by two researchers (LS and MCP). To gain familiarity with the content, the transcripts were read several times. Each unit of text was then coded, a code being defined as a meaningful unit describing a section of text (for example, the code “Helping patient’s self-evaluation” described the following text section: “With a self-administered survey on the app, the patient could do self-assessments”). Codes were organised into categories (for example: “data provided by the app”, including codes such as “exercises” or “therapeutic tools”) and themes (for example, “content of the app”, including categories such as “features” or “data collected by the app”). The codes, categories and themes were compared and agreed upon among the research team. In the case of discrepancies between researchers, agreements were reached by individually clarifying the meaning of a code and discussing its interpretation until mutual consent was achieved. If necessary, the codes, categories and themes were updated*.* Team meetings were held to discuss and monitor coding consistency and to address the analytic validity of the identified themes. Moreover, the research team met to ensure that the findings were internally consistent and supported by the data from the participants’ interviews. After four patient focus groups and four physician focus groups, no new codes or categories were emerging, indicating the reaching of data saturation.

Patients and physicians respective focus groups have been analysed separately to identify the discrepancies between them. The two codebooks were then merged into a single codebook.

Sociodemographic data of the sample are presented as the mean (Standard Deviation, SD) for continuous variables and frequency distribution for categorical variables.

## Results

### Participant characteristics

The sample’s general characteristics are summarised in Table 1. A total of 24 patients and 26 physicians were included in the study.

**Table 1:** Sample characteristics (*n* = 50)

**Patient characteristics (*N*** **= 24)**	**Mean ± SD or n (%)**
Male	13 (54.1)
Age	51.5 ± 15.5
Urban living	11 (45.8)
IDS-SR score	36.9 ± 12.2
Phone owners	24 (100.0)
Smartphone owners	20 (83.3)
Android /IOS	18 (75) / 2 (8.3)
App users	19 (79.2)
App number	16.2 ± 19.0
Health App number	0.3 ± 0.6
**Physician characteristics (*N*** **= 26)**	**Mean ± SD or n (%)**
Male	13 (50.0)
Age	45.5 ± 12.2
Urban practice	24 (92.3)
General Practitioners	10 (38.5)
Private practice	13 (50.0)
Years of practice	15.8 ± 11.6
Visits per week	66.8 ± 50.0
Patients with depression seen per week	14.0 ± 11.0
Phone owners	26 (100.0)
Smartphone owners	25 (96.1)
Android/IOS	14 (53.9) / 11 (42.3)
App users	25 (96.1)
App number	28.9 ± 29.0
Health App number	2.8 ± 3.1

*SD* standard deviation, *IDS-SR* inventory of depressive symptomatology, *IOS* iphone operating system

For patients, the focus groups showed balanced sex (male/female ratio = 13/11) and age distributions (from 20 to 73 years). The mean ± SD age was 51.5 (± 15.5). Most of the patients had a smartphone (83.3%) and were app users (79.2%).

For physicians, the focus groups also showed balanced sex (male/female ratio = 13/13), age (from 31 to 67 years) and type of practice distributions (private/public ratio = 13/13). The mean ± SD age was 45.5 (± 12.2). Most of the physicians had a smartphone (96.1%) and were app users (96.1%).

### Identification of themes

All data were collected during the focus groups. Within-group consensus was rare, as the point of qualitative research is to highlight all the opinions and not to find a consensual one.

A content analysis of the verbatim data resulted in primary codes, which, after an inductive interpretation and categorisation process, were structured within three themes (Table 2):

1. Content of the app
2. Operating characteristics
3. Barriers to the use of the app

**Table 2:** Analysis codebook

Theme	Categories
Content of the app	Data collected by the app
	Data provided by the app
	Features
Operating characteristics	Relevant population
	Aims of the app
	Modalities and frequency of use
	Accessibility and visibility
	Security
Barriers to the use of the app	Users and accessibility
	Safety and side effects
	Utility and functioning

### Theme 1: content of the app

#### Data collected by the app

 Patients and physicians felt that an ideal app should collect data about its users to ensure the delivery of a suitable content. They mentioned the creation of a profile for the patient using the app, with medical history and general information, including their interests and leisure activities.

Patients and physicians agreed on the need to assess symptoms. Opinions diverged on the way to do it, some considering the use of validated questionnaires and scales and others showing a preference for free texts or logbooks. Mood charts were described by patients and physicians as useful items. It was considered a good method to collect symptom descriptions, including their intensity, chronology, rhythm, evolution and their impact on patients’ quality of life. In addition to the collection of symptoms, both patients and physicians mentioned the benefits of checking medication uptake, allowing a close monitoring of medication compliance.

*“With a self-administered survey on the app, the patient could do self-assessments. He could follow his clinical status and this could help him to realise ‘I feel better than last week.’”* Physician 1, group 3.

Among participants, physicians only discussed the interest of using passive phone data (data collected without the active action of the patient; i.e. the number of steps a day…), considered as a desirable feature in order to collect reliable information on patients’ state without active participation. They were of the opinion that this approach would overcome patients’ subjective assessments in order to obtain reliable and accurate data. Some physicians, however, were sceptical of this intrusive process and raised its possible non-acceptance by patients.

*“Collection of passive data. It is the most obvious interest point. […] If we can get data from SMS and other messaging apps, we will have, in the end, really precise clinical indicators. The only comparable thing would be to discuss with a caregiver living with the patient 24/7.”* Physician 4, group 1.

#### Data provided by the app

Patients and physicians suggested several tools that help them in daily life that should be integrated within the app. They included a diary to keep appointments and a phonebook to facilitate communication with healthcare professionals and associations.

Information about depression was also considered, organised in different sections: a clinical section with symptom descriptions, a medication section with therapeutic strategies, drug interaction and potential side effects, and a social section to help them with administrative tasks. The lack of knowledge about the disease has been pointed out to explain this need for information about depression. Furthermore, patients and physicians showed a real expectation of professional advice in various domains such as therapeutic adaptation in crisis or alternative medicines.

*“I would like to know, considering our usual medication, if we can take other drugs. Me for example, I take lithium, and with lithium, you cannot take, for example, non-steroidal anti-inflammatory drugs. So, maybe there are other drugs we cannot take?*” Patient 1, group 3.

Patients and physicians also cited several exercises and therapeutic tools they would like to find in this app such as games, relaxation sessions or cognitive-behavioural therapy exercises. They explained that this kind of tool could be an easy way to extend the work initiated with the therapist in face-to-face visits. They also agreed on the necessity of specific content for physicians, including patient profiles, clinical and therapeutic recommendations based on clinical practice guidelines and a messaging service. This content could support physicians to optimise patient care and sustain interactions between patients and therapists, thus providing feedback regarding patients’ mental states through the use of the app.

“*From the psychiatrist side, if I had to have such an app, I’d like to have therapeutic help finding the next line of treatment. The app could use the previous treatment of the patient and even his medical history to determine the best medication to use next.”* Physician 4, group 3.

Among participants, only patients asked for other patients’ testimonies, explaining they could feel comforted by reading stories from other people struggling with depression. They also expressed a need for a medical chat room to receive reliable and adapted advice.

#### Features

Patients and physicians stated that the app should be tailorable. Personalisation was seen as the best way to obtain an app suitable for the greatest number of people that is able to adapt to patients’ clinical states and their changes.

Various technical features were suggested, such as automatic replies by notifications or SMS or through a virtual interlocutor (i.e. chat bot). Patients and physicians considered that the app could also use and collect data provided by other connected tools (e.g. wearables) to improve its capacity to assess patients’ symptoms in momentary ecological conditions.

Charts were described as being useful to display symptoms collected by the app. The use of different colours and smileys was also cited as a smart method of highlighting important information and giving the app a friendly aspect.

*“A diagram or a histogram, well visible, easy to read. We need something more ludic, intuitive and easy*.” Physician 1, group 2.

Dealing with emergencies was a feature judged to be essential to an app dedicated to depression by both patients and physicians. Different features were considered for this purpose, from written advice provided by the app to a call to their physician or family. Patients and physicians agreed on the utility of a partnership with emergency services to ensure a quick and reliable reaction to a suicidal crisis.

*“The app could send automatic replies in* [the] *case of symptoms worsening. It could be advice or suggestions. In* [the] *case of suicidal thoughts or immediate risk, the app could directly call the patient’s physician.*” Physician 6, group 1.

However, physicians expressed some concerns about emergency management by the app, as developed in the “security section”. These concerns were not found in the patients’ groups.

### Theme 2: operating characteristics of the app

#### Relevant population

Participants were invited to discuss an app dedicated to patients with depression; nonetheless, they emphasised the need for a tool dedicated to non-diagnosed patients. To facilitate and increase mental healthcare access, they suggested an app to screen, among the general population, people with a feeling of unease.

*“‘ …Do I feel good?’ I think it could be interesting to help people to ask themselves this question before seeing a professional. Actually, patients know nothing about depression. They think: ‘I’m just tired, I’m gonna rest and everything will be alright,’ and* [a] *depression diagnosis is made really late. […] Today people struggle with depression until they are exhausted. They don’t have the opportunity to assess themselves. They don’t find an app to know if they suffer from depression.”* Physician 3, group 2.

Patients and physicians also agreed on the necessity of an app, or a section of the app, dedicated to patients’ caregivers, seen as an essential actor in depression care. They pointed out caregivers’ usual lack of knowledge about depression and suggested that an app with information about depression and an indication of what they should do in the case of relapses could be useful.

Regarding the target users of an app dedicated to depression, patients and physicians pointed out the risk of difficulties in its implementation and use with patients suffering from severe depression. They considered that it should be preferable for patients suffering from mild to moderate depression.

Finally, patients and physicians cited psychiatrists and general practitioners among healthcare professionals who could be connected and linked to patients’ accounts.

#### Aims of the app

Patients and physicians suggested the app could have different aims. These aims vary according to the targeted users and their clinical state.

The four main objectives were screening, orientation, information and monitoring. Screening and orientation were associated with apps dedicated to the general population, which could diagnose depression and refer patients to a mental healthcare professional. Information was considered in two ways: first, to sensitise the general population to depression and to destigmatise it; secondly, within a psychoeducational perspective, for patients suffering from depression. Monitoring was considered to overcome the memory bias encountered in monthly follow-ups, with patients often remembering how they felt for the few days preceding the visit or focusing on the worst state they have encountered in that month, regardless of how long it had lasted.

*“Sometimes in visits you’re asked, ‘How are you doing? What happened since the last time?’ And you have forgot! A useful thing could be a questionnaire to check your mental state and feelings everyday so you can show it to your physician on the next visit.”* Patient 5, group 3.

The app was also considered by patients and physicians to be a reassurance tool, providing a presence during weekends and physicians’ vacation time.

Physicians suggested other objectives not mentioned by patients. They considered the app to be of additional value in depression care, being an intermediate between them and their patients, allowing access to complementary information and facilitating the discussion of personal topics in visits, such as sexuality. It was also described as possibly promoting care adherence and reducing anxiolytic use through therapeutic education.

On the other hand, they only insisted on the creation of a social network allowed by the app, proposing a forum and chat feature for patients to share their experiences with their peers.

#### Modalities and frequency of use

Opinions on the modalities and frequency of app use were numerous and strongly diverged among participants.

The optimal frequency of use ranged from daily to bi-weekly or only on the patient’s request. Where some patients or physicians considered unlimited use of the app, others preferred to restrict its use to depressive episodes only.

“*I think this app should have* [an] *unlimited lifetime; it shouldn’t stop. Even if it means we have to deactivate some features with time…”* Physician 2, group 4.

Only physicians discussed the possibility of naming a trusted person among the patient’s entourage whose contact information (phone or email) would be registered in the app. The trusted person could be contacted (i.e. an alert by mail or SMS) by the app in case of an emergency or a reminder with their phone number could be displayed to make it easier for the patient to call them when they are feeling unwell. They also suggested the reception of patient data should occur during working hours only. Some even suggested data should only be shared during patient visits and that physicians should only be contacted through the app in the case of an emergency. To facilitate the use of the app and help them to gain time, data transfers to physicians’ medical software were also considered.

“*When I see the patient in visit*[s]*, I could log on the patient’s app. I could even transfer what has been measured by the app on my medical software.*” Physician 5, group 1.

Conversely, patients insisted on the need to be able to reach their physicians, or at least a mental healthcare professional each time they needed to, regardless of the time or day.

#### Accessibility and communication around the app

Both patients and physicians felt that access to the app should be easy. They suggested it could benefit from good visibility on the web and be presented to patients through educational material available in physicians’ waiting rooms.

*“So, when you write ‘Am I depressive?’ on an internet search engine, the first result should be this app, and you just have to click on it to download it.”* Physician 1, group 2.

Access to the app was a dividing point, with participants in favour of unrestricted access in app stores and others suggesting an app available after medical prescription only.

Patients and physicians emphasised the need for a free app to ensure access for all patients with depression, regardless of their incomes.

#### Security

The app’s data security and privacy policies were discussed as being a fundamental requirement in both patient and physician groups. They agreed on access protected by a password or code. Data storage was a point of concern; participants considered that such a device requires dedicated secured servers to store and protect collected data.

“*It’s problematic, we need storage* [on a] *secured server.*” Physician 2, group 4.

As mentioned in the “features” section, physicians but not patients considered an app that would work on patients’ phones only, with no professional dashboard, assuring strict confidentiality and limited access to data.

Physicians also insisted on medico-legal issues raised by mental health information transmission and emergency management. They emphasised the need for well-defined conditions of data transmission to physicians, keeping in mind that they would not check the app during nights or weekends and so there may be delays in emergency situations.

“*The app could raise some medicolegal issues. If the patient tries to contact us, it's the same with e-mail, patients could believe or think we have received and processed his message. If we don’t receive it, or* [we receive it] *too late, we could* [be] *held medically responsible.*” Physician 4, group 4.

### Theme 3: barriers to the use of the app

#### Users and accessibility

Patients and physicians showed themselves to be sceptical about the ability of a patient to use the app during a depressive episode, regardless of severity. They insisted on the potential impact of depressive symptoms on the use of the app, such as cognitive dysfunction and anhedonia.

Costs and material access were pointed out as limitations of the democratisation of the app, making it a tool restricted to a privileged population. Patients and physicians also suggested that this kind of device could not be applied to all age groups, with the risk that older patients may struggle with the use of new technologies.

Only physicians described the risk of time consumption associated with the use of the app as a strong limitation. They worried about not being able to deal with the app in addition to their other professional duties. They also expressed doubts about their ability to integrate these tools into their daily practice.

*“Let’s take email: I read it once every two days. I cannot check it more often. I come back home really late, 11.30 pm sometimes. Checking mail and SMS and answering it takes a lot of time. Honestly, I don’t think I could answer patients contacting me through an app.”* Physician 2, group 3.

#### Safety and side-effects

Patients and physicians worried about potential side effects related to the use of the app. Anxiety was seen as a symptom that could be worsened or created by the app. Overuse of the app and social withdrawal were also suggested to be potential side effects. Participants assumed that the daily use of an app would keep users away from their relatives and so increase withdrawal symptoms already seen in depressive disorders.

The risk of a decrease in visit frequency with healthcare professionals was suggested, as well as a progressive replacement of the physicians by the app, both in evaluation and therapeutic aspects. Patients and physicians argued that the app could provide the therapist with a feeling of security and encourage them to space appointments out. They were particularly concerned about the risk of reducing the number of face-to-face visits, meaning for them a loss of human contact, considered as essential in depression care.

“*Then I think we should be careful with these medical apps because in the end it will promote telemedicine: if all indicators are positive, the patient takes its treatments, he displays happy smileys, why* [should we see] *him in visits?”* Physician 1, group 4.

#### Utility and functioning

Among participants, some patients and physicians showed themselves to be sceptical about the utility of such an app. They expressed that they could not see any utility in it, thinking that the use of a connected device for psychotherapy is nonsense. They also emphasised the already wide range of apps available for depression and explained a new app would not improve care for patients with MDE.

Some physicians suggested that psychosocial interventions such as psychoeducation could not be done without healthcare professionals and expressed doubts about patients’ comprehension of information delivered by the app.

“*The issue is* [the] *interpretation of messages received by the patient. You don’t have an instantaneous feedback for it and if he had a wrong interpretation it will not help him.*” Physician 3, group 2.

“*I think that psychiatry is based on discussion, trust, empathy, everything that makes medicine an art, and I think all this cannot pass through an app.*” Physician 2, group 2.

Only patients expressed doubts about the potential marketing use of the app, and stated that advertisements would be unwelcome while using the app and would make them uncomfortable.

## Discussion

This qualitative study is the first to assess both physician and patient expectations of an app dedicated to depression. The use of the focus group method provided a range of experiences and opinions among the participants and a relationship of trust among the group. The discussions it allows increased the role of the participants who collectively built the results of the research.

All themes and categories were the same for patients and physicians, highlighting a shared interest and mutual needs regarding this tool. However, some code differences pointed out the potential conflict between patients’ needs and physicians’ constraints. Direct access to a professional through the app, which was a strong wish expressed in the patient group, was not mentioned in the physician group and echoed the worry about physicians’ availability to use an app. Nevertheless, these conflicts remained scarce and this study revealed strong similarities between patient and physician expectations, revealing that an app suitable for both patients and physicians could easily be developed. Patients and physicians expressed expectations regarding its content, its operating characteristics and discussed potential barriers to its implementation in real-world clinical practice. Content considered by participants included data provided to and delivered by the app, as well as features thought to be useful for that kind of tool. Data collection methods must gather information about the patient using the app to evaluate their mental state and to inform physicians of the evolution of depressive symptoms. Data delivered by the app should provide psychoeducation elements, therapeutic tools and various functionalities to aid the management of daily life. Features considered for the app were meant to facilitate its use by physicians and ensure patient care in the case of an emergency. The “operating characteristics” theme showed strong heterogeneity between participants’ expectations regarding target users, frequency of use and aims of the app. This heterogeneity emphasised the interest of a tailorable tool to meet all the needs and desires of patients and physicians. Finally, this study highlighted doubts and limitations that both patients and physicians may have regarding an app dedicated to depression. These barriers included concerns about users of the app, its accessibility, safety, side effects, utility and functioning.

Most of our results are consistent with previous qualitative studies on health apps, whether they focus on apps for depression or not. Regarding content, information on the disorder and day-to-day life-supporting activities like music, breathing exercises and videos are often cited as expected items [27, 32, 33]. Self-tracking is a highly rated activity of health apps [27, 32] and is described by patients as a reason to return to the app [34]. Similarly to our findings, among possible app features, personalisation is the most requested and liked, both by patients and professionals [26, 27, 32–35]. The expected aims of such apps found in the literature included psychoeducation [35], monitoring [27, 32, 35], providing a presence between face-to-face visits [27] and social connectivity [26, 32]. Consistently with our result, other studies highlighted two main barriers to the use of apps cited by physicians: lack of time and medicolegal responsibility [27, 28]. Financial aspects and deficiency in technological competencies are common barriers to the use of apps for patients [26, 33, 35].

An important issue considered by the participants in our study was the type of population that could benefit from an app dedicated to depression. First, in line with other studies, patients and physicians agreed to focus on an app for people suffering from mild-to-moderate depression [24]. This statement tended to be confirmed by a recent meta-analysis focusing on the efficacy of app interventions for depressive symptoms, the post hoc subgroup analysis showing that significant benefits from smartphone apps were only found for patients with self-reported mild-to-moderate depression [36]. Those results should be considered carefully, with the variations in subgroup sample sizes leaving the analyses for major depression underpowered to detect significant effects. Moreover, a more recent study identified that more severe depression led to enhanced information seeking, counteracting the theory that severe depression keeps patients from using apps [37].

An app to screen potential depression in the general population was also discussed. Participants emphasised the significant role that this app could provide in facilitating access to mental health for depressed people. They assumed that the large-scale use of such an app could decrease the mean duration of untreated depression and hence the recurrences and the duration of MDEs [38, 39]. In line with our results, several studies pointed out the possible interest of apps for depression screening in the general population, showing that a large number of people from different countries were searching for, and willing to use, that kind of tool [40]. Additionally, several apps using text analysis have shown their ability to improve the immediate detection of depressive symptoms [41, 42]. Finally, several studies highlighted the fact that depression screening apps could motivate some users to discuss the obtained results of the tests with healthcare professionals for further diagnosis and management [43, 44].

Our findings also emphasised the interest in an app that would not be time-consuming for physicians and could help treatment decision-making in patients with depression based on the most updated and high-quality evidence. To the best of our knowledge, there are very few apps currently available providing evidence-based guidance for treatment decision-making to physicians.

One study highlighted that an app could be an effective tool for both increasing confidence in depression treatment and educating physicians [45], pointing out the interest to develop more connected tools for healthcare professionals.

Finally, participants mentioned the usefulness of an app for informal caregivers, to inform them, help them in supporting their ill relative and to destigmatise mental health. This demand is supported by the results of a recent systematic review focusing on apps dedicated to caregivers, showing most of the included studies proved their effectiveness in the overall well-being of the caregivers [46]. Apps dedicated to caregivers, who are often suffering themselves from depression or anxiety, could significantly improve mental healthcare regarding their essential role for patients suffering from depression [47].

These findings highlight that a single app is not enough: multiple versions of the app are needed to encompass the support and care objectives of patients, mental health professionals and informal caregivers.

One crucial feature expected by participants was self-monitoring. The main interest of it is to improve mental health and wellbeing by increasing emotional self-awareness [48, 49]. The use of apps for self-monitoring allows precise, easy and quick ecological momentary assessment with the possibility of providing real time feedback for patients and alerts for clinicians in case of emergencies. It would also allow clinicians to monitor the efficacy of treatment over time, predict short-term mood changes and detect the worsening of symptoms early on [50]. There are many apps for depression integrating a self-monitoring feature and several studies have examined their usability, acceptability, adherence and effectiveness. Self-monitoring of depressive symptoms on patients’ phones has been shown to be easy and reliable [51, 52] and several studies highlighted its effectiveness to improve depressive symptoms [36, 53, 54]. A study on untreated patients with symptoms of depression and anxiety also showed that access to daily self-monitoring helped them to translate their intention to seek treatment into actual treatment-seeking behaviour [55]. Even if this effect was small, it defines self-monitoring as a promising tool to decrease the number of untreated patients suffering from depression. However, the main bottleneck of self-monitoring is the low retention rate of apps offering this feature. For this particular point, studies showed inconsistent results, where some highlighted a quick drop out rate of self-monitoring apps [56, 57], while others noted, conversely, a fairly high retention rate for these tools [52, 54, 58].

In addition to self-monitoring and patients’ active input of data, passive data collection has been suggested by physicians in our study. This is consistent with the new research allowed by advanced technologies such as digital phenotyping [59], aiming to determine clinical phenotypes by measuring patient behaviours from smartphone sensors [60]. Requiring no active participation from the patient, the collection of passive data has been increasingly studied in recent years and seems to be a promising field for the future of m-health. This method could indeed allow ecological momentary assessment of several parameters and could be a strong tool to improve depression care. An interesting application domain for this method is the use of an algorithm to facilitate the detection of new MDEs that could be of significant help for clinicians in the follow up of patients with mood disorders [61–64]. A study focusing on therapists’ ability to detect negative changes in their patients showed that clinical judgement allowed the detection of only 21% of symptom worsenings [65]. Early detection of an episode with passive data collection could then facilitate the quick reaction of physicians and improve patients’ outcomes [66]. Despite being promising, passive data collection is, as shown in our study, not yet accepted by everyone, whether patients or physicians, and remains a strong barrier to the use of apps.

The analysis of the barriers identified the potential replacement of the physician by the app as a major concern in psychiatry, where state-of-the-art methods require human interaction. This barrier is raised in several studies who mentioned the lack of therapist contact as a negative point of apps [24, 34, 67]. This issue could be overcome with the use of mixed methods or adjunctive apps, integrating the app in face-to-face therapy. This kind of approach has shown better efficacy than self-guidance therapy only through web intervention or smartphone apps [68–71], thus demonstrating the essential role of the therapist in patient care. All these elements emphasised an essential point: complementarity between therapists and m-health tools. In Western countries, the main aim of these devices is to support existing care by providing, for example, better information regarding the patient’s daily state through momentary ecological assessment. The use of new technologies should not be seen as a replacement tool for physicians but as an opportunity to increase the quality of care provided. The inclusion of apps in therapy could then be compared to the development of imagery devices in radiology, improving diagnosis without removing the need for clinical examination and physicians’ knowledge and expertise.

### Perspectives

The key findings of this study allow us to build a list of suggestions for app developers in the field of depression to fill both patients’ and physicians’ expectations:

- The use of the app should be easy and intuitive.
- The app should be personalised. The content and functioning of the app should be tailored to each patient and should adapt to the patient’s condition over time.
- A self-monitoring function should be included to both increase the patient’s self-awareness and sharpen the evaluation of the physician. This function should focus on key symptoms and offer the patient and the physician the possibility of choosing other symptoms to monitor.
- The app should be able to deal with emergency situations. At the least, it should include a tailorable crisis procedure and, at best, include a partnership with the local emergency service.
- The app should provide the patient with information about depression and/or psychoeducational messages.

Furthermore, our results highlighted several key points that should remove the potential barriers to the use of the app:

- Relevant users should be precisely targeted to ensure their capacities to use the app, with particular attention given to the intensity of depression.
- To reduce the potential difficulties bound to the use of the app, physicians should plan a dedicated educational time with the patient to explain the functioning of the app. A user guide should also be included and delivered to the patient.
- Mixed approaches should be preferred. The app should be fully integrated with the usual therapy and be an adjunctive tool rather than an independent one. The use of the app should not lead to any reduction of the frequency of visits to the therapist.
- The use of the app should be free of charge.
- Access should be protected by a password and data stored in a secured server.

### Strengths and limitations

This study has a number of limitations. Selection bias may have occurred: patients’ recruitment was limited to France for practical reasons. Furthermore, most of the physicians practiced in urban areas. Therefore, the findings may not be transferable to practitioners in rural areas. This study was also limited to some degree in the use of *focus groups* as a methodology: group dynamics might have, in some way, shaped the expectations expressed by participants and the interviewers’ personal skills and attributes could also have influenced the nature and quality of the gathered data. However, the use of focus group is also one of the strengths of this study as it allows for interaction among participants and facilitates discussion and sharing of ideas.

## Conclusion

Physician and patient expectations of a smartphone app dedicated to depression are significant, suggesting a real place for such a device in the management of depression. The key points expected by the users for such a tool are an easy and intuitive use and a personalised content. They are also waiting for an app that gives information about depression, offers a self-monitoring functionality and help them in case of emergency. To ensure good implementation and retention rates, these expectations must be of major concern while developing these tools. Finally, apps should be considered as medical devices and be tested in clinical trials. Consequently, their development requires further studies to ensure their efficacy and safety.

## Supplementary Information

**Additional file 1.** Interview guide. Details of the interview guide used to facilitate discussion within the focus groups.

## Data Availability

The datasets used and/or analysed during the current study are available from the corresponding author on reasonable request.

## Abbreviations

MDE: Major depressive episode
FG: Focus group
DSM-IV-TR: Diagnostic and statistical manual of mental disorders - 4th edition, text revision
IDS-SR: Inventory of depressive symptomatology
SD: Standard deviation
CBT: Cognitive-behavioural therapy
