# Supplementary material for: Patient and physician perspectives of a smartphone application for depression: a qualitative study
Source: BMC Psychiatry. 2021 Jan 29;21:65. doi: 10.1186/s12888-021-03064-x (PMC7847000; doi:10.1186/s12888-021-03064-x)
Supplement: Supplementary file 1 — Additional file 1. Interview guide. Details of the interview guide used to facilitate discussion within the focus groups. [file 12888_2021_3064_MOESM1_ESM.docx]

**Patient and physician perspectives of a smartphone application for depression: A qualitative study**

Marie-Camille Patoz^1^, Diego Hidalgo-Mazzei^2^, Olivier Blanc^1,3^, Norma Verdolini^2^, Isabella Pacchiarotti^2^, Andrea Murru^2^, Laurent Zukerwar^4^, Eduard Vieta^2^, Pierre-Michel Llorca^1,3^, Ludovic Samalin^1,3,*^

^1^ Department of Psychiatry, CHU Clermont-Ferrand, University of Clermont Auvergne, EA 7280, Clermont-Ferrand, France

^2^ Bipolar and Depressive Disorders Unit, Hospital Clinic, University of Barcelona, Institute of Neuroscience, IDIBAPS, CIBERSAM, 170 Villarroel st, 12-0, 08036, Barcelona, Catalonia, Spain

^3^ Fondation FondaMental, Hôpital Albert Chenevier, Pôle de Psychiatrie, Créteil, France

| Additional File 1: Interview guide |  |
| --- | --- |
| **Patients’ interview guide** | **Physicians’ interview guide** |
| **Q1. How could an app help you when you experience depression symptoms?** | **Q1. How could an app be an interesting tool for patients with MDE?** |
| Q1.1How could an app help you with medication? | Q1.1 How could an app designed for patients with MDE help them? |
| Q1.2 How could an app be useful to collect information on your symptoms? Which information? | Q1.2 What kind of information could be useful to collect about patients’ symptoms? |
| Q1.3 How could an app be useful to ease your return to some activities? How? | Q1.3 How could an app help patients with MDE regarding medication? |
| Q1.4. How could an app be useful to enhance your pathology knowledge? How? | Q1.4 How could an app be useful to ease MDE patients’ return to physical activity? |
|  | Q1.5. How could an app be useful to enhance the pathology knowledge of patients with MDE? |
| **Q2. How do you imagine this app in practice?** | **Q3. How do you imagine this app in practice?** |
| Q2.1. How should its content be displayed? | Q3.1. How should its content be displayed? |
| Q2.2. Should this app be secured or follow any regulatory framework? | Q3.2. How must this app be secured? |
| Q2.3. In what form would you like to receive information thanks to this app? | Q3.3 In what form could information be communicated to the patients thanks to this app? |
| Q2.4. How do you think you would use this app in terms of duration? | Q3.4 How should this app be used in terms of duration? |
| **Q3. How could an app help your physician when you experience depression symptoms?** | **Q2. As physicians, what interest do you see in an app dedicated to MDE?** |
| Q3.1 What kind of information could your physician receive? | Q2.1. In what way could an app help YOU, as a physician, in your daily practice with these kind of patients? |
| Q3.2 When could this information be transmitted to your physician? | Q2.3. How would you like this information to be transferred to you? |
| Q3.3. Do you wish an alert could be triggered from this app when you feel really bad? How? To who? | Q2.4. When could this information be transferred to you? |

^4^ Clinique Mon Repos, Ecully, France

* Corresponding author: Ludovic Samalin, Centre Hospitalier et Universitaire, Service de Psychiatrie B, 58 rue Montalembert, 63000 Clermont-Ferrand, France. Tel: +33 473 752 124, Fax: +33 473 752 126. *E-mail address*: [lsamalin@chu-clermontferrand.fr](mailto:lsamalin@chu-clermontferrand.fr)
